# The Aldosterone-Mineralocorticoid Receptor Pathway Exerts Anti-Inflammatory Effects in Endotoxin-Induced Uveitis

**DOI:** 10.1371/journal.pone.0049036

**Published:** 2012-11-09

**Authors:** Elodie Bousquet, Min Zhao, André Ly, Guillaume Leroux les Jardins, Brigitte Goldenberg, Marie-Christine Naud, Laurent Jonet, Bernadette Besson-Lescure, Frederic Jaisser, Nicolette Farman, Yvonne De Kozak, Francine Behar-Cohen

**Affiliations:** 1 INSERM U872, Université René Descartes Sorbonne Paris Cité, Team 17, Centre de Recherche des Cordeliers, Paris, France; 2 Université René Descartes Sorbonne, Paris Cité, France; 3 Assistance Publique des Hôpitaux de Paris, Hôtel-Dieu, Paris, France; 4 Plateforme technologique de phénotypage du petit animal et microdosages. IFR65/IRSSA, Hôpital Saint-Antoine, Paris, France; 5 INSERM U872, Université Pierre et Marie Curie, Team 1, Centre de Recherche des Cordeliers, Paris, France; Oregon Health & Science University, United States of America

## Abstract

We have previously shown that the eye is a mineralocorticoid-sensitive organ and we now question the role of mineralocorticoid receptor (MR) in ocular inflammation. The endotoxin-induced uveitis (EIU), a rat model of human intraocular inflammation, was induced by systemic administration of lipopolysaccharide (LPS). Evaluations were made 6 and 24 hours after intraocular injection of aldosterone (simultaneous to LPS injection). Three hours after onset of EIU, the MR and the glucocorticoid metabolizing enzyme 11-beta hydroxysteroid dehydrogenase type 2 (11β-HSD2) expression were down-regulated in iris/ciliary body and the corticosterone concentration was increased in aqueous humor, altering the normal MR/glucocorticoid receptor (GR) balance. At 24 hours, the GR expression was also decreased. In EIU, aldosterone reduced the intensity of clinical inflammation in a dose-dependent manner. The clinical benefit of aldosterone was abrogated in the presence of the MR antagonist (RU26752) and only partially with the GR antagonist (RU38486). Aldosterone reduced the release of inflammatory mediators (6 and 24 hours: TNF-α, IFN-γ, MIP-1α) in aqueous humor and the number of activated microglia/macrophages. Aldosterone partly prevented the uveitis-induced MR down-regulation. These results suggest that MR expression and activation in iris/ciliary body could protect the ocular structures against damages induced by EIU.

## Introduction

The aldosterone/mineralocorticoid receptor (MR) pathway is known for its role in extracellular volume and blood pressure regulation through the renal sodium absorption and potassium excretion in the distal nephron epithelium. Aldosterone binds to the mineralocorticoid receptor (MR), a transcription factor of the steroid receptor superfamily that also includes the glucocorticoid receptor (GR) [1], [2]. The MR binds aldosterone and glucocorticoids with similar high affinity. Glucocorticoids largely prevail in the plasma. Permanent MR occupancy by glucocorticoids is prevented by the metabolizing enzyme 11-beta hydroxysteroid dehydrogenase type 2 (11β-HSD2), which transforms the glucocorticoids (cortisol in humans, corticosterone in rodents) into metabolites that have a weak affinity for the MR [1], [2].

Mineralocorticoid specific effects require co-expression of MR and 11β-HSD2 like in the distal nephron. Beside this classical role in the kidney, the aldosterone/MR pathway was discovered to exert regulatory functions in non-epithelial cells including neurons, adipocytes, cardiomyocytes and in vascular cells [1], [2]. Pathological MR activation has been evidenced in cardiovascular diseases [3]. Indeed, its antagonism by anti-mineralocorticoid drugs (spironolactone, eplerenone) has proven to be mostly beneficial to limit morbidity and mortality in heart failure and myocardial infarction [4], [5]. Pathological chronic MR activation is suspected to induce tissue damages at least in part through inflammation [6]. In myeloid cells, MR activation was shown to favor M1 macrophages polarization whilst MR abrogation induced M2 polarization and subsequent cardiovascular tissue protection [7]. In the brain, acute post-ischemic damages were also reduced in myeloid-MR deficient mice [8]. Moreover, in patients with diabetic nephropathy, MR blockade reduced inflammation markers [9], [10]. Aldosterone increased the expression of vascular cell adhesion molecule-1 in aortic endothelial cells [11] and stimulated a pro-inflammatory phenotype in other non-inflammatory cells such as adipocytes [12] and smooth muscle cells [13] reinforcing the idea that MR pathway activation in both resident and inflammatory cells stimulates inflammation, contributing to subsequent tissue damage.

We have recently shown that retinal cells and choroidal endothelial cells express MR and 11β-HSD2, identifying the retina and the choroid as mineralocorticoid-sensitive tissues [14], [15]. The MR and the 11β-HSD2 are also expressed in the iris and ciliary body [16], [17], which are part of uveal tract, the mid-layer of the eye (Fig. S1). These tissues are targets of ocular inflammation called uveitis. In humans, intraocular inflammation can be idiopathic or part of systemic inflammatory diseases such as spondyloarthritis or Behçet’s disease [18]. Uveitis is responsible for about 10% of the cases of severe visual handicap [18]. In the eye, blood-ocular barriers contribute to protect ocular tissues from inflammatory cells invasion and subsequent tissue damages [19]. Blood-aqueous barrier is formed by tight-junctions between endothelial cells of the iris and between epithelial cells of the ciliary body [20]. Uveitis is associated with ocular barrier breakdown through mechanisms that are not fully understood.

The endotoxin-induced uveitis (EIU) is a well-characterized rat model for human acute uveitis [21]. Resident cells (microglial cells, iris/ciliary body endothelial and epithelial cells) are activated early and produce cytokines, chemokines and iNOS (inducible nitric oxide synthase) about 4 hours after LPS injection [22]. Later (8 hours), circulating inflammatory cells (polymorphonuclear leukocytes and macrophages) invade the anterior segment tissues of the eye [22], [23], [24].

The implication of aldosterone/MR pathway activation in ocular inflammation has never been questioned. Our aim was to evaluate the impact of MR pathway activation or inhibition on EIU. Unexpectedly, aldosterone exerted a beneficial anti-inflammatory effect.

## Materials and Methods

### Animals

Animal experiments were approved by the Institutional Animal Care and Use Committee: «Comité d’éthique en expérimentation animale Charles Darwin» (ID Ce5/2009/029), INSERM U289, Hôpital Salpétrière, Paris and performed in accordance with the ARVO Statement for the Use of Animals in Ophthalmic and Vision Research. Adult female Lewis rats (6–8 weeks old; Janvier, Le Genest-Saint-Isle, France) were used (number of animals (n) are indicated in Fig. legends). Anesthesia of rats was induced by intraperitoneal injection of pentobarbital (25 mg/kg Nembutal; Abbot, Saint-Remy sur Avre, France). One drop of 1% tetracaine (Sigma-Aldrich, Saint Quentin Fallavier, France) was instilled for local eye anesthesia before intravitreal injection. Rats were sacrificed by a lethal dose of pentobarbital intraperitoneal injection. After sacrifice, the eye was removed, either processed for morphology or dissection to collect aqueous humor and iris/ciliary body.

### Induction of EIU

EIU was induced in rats by a single footpad injection of 100 µl sterile pyrogen-free saline containing 200 µg lipopolysaccharide (LPS) from Salmonella typhimurium (Sigma-Aldrich) as previously described [25].

Acute anterior segment inflammation of the eye reaches its maximum intensity at 24 hours, then spontaneous resolves in 7 days [21], [22].

### Aldosterone and Spironolactone Injections into the Vitreous Body of the Rat Eye

Aldosterone or MR antagonist (spironolactone, RU26752) or GR antagonist (RU38486) or solvents (NaCl 0.9% with 0.1% ethanol) were injected into the vitreous body of both eyes (intravitreal injections: IVT) as previously described [15] simultaneously to LPS injection. We injected 5µl (i.e. 10% of the vitreous volume) of aldosterone (1 nM, 20 nM or 200 nM final concentrations) or spironolactone (10 µM), or MR antagonist RU26752 (10 µM), or GR antagonist RU38486 (10 µM), or aldosterone (200 nM) associated with the MR antagonist RU26752 (20 µM), or aldosterone (200 nM) associated with the GR antagonist RU38486 (20 µM). Drugs were purchased from Sigma-Aldrich.

### Clinical Examination

The inflammation was scored 24 hours after LPS challenge and intravitreal injection using biomicroscopy analysis as previously described [25]: grade 0: no inflammation; grade 1, minimal iris and conjunctival vasodilation but without cells in the anterior chamber; grade 2, moderate iris and conjunctival vessel dilation but without evident cells in the anterior chamber; grade 3, intense iris vessels dilation and fewer than 10 cells per slit lamp field in the anterior chamber; grade 4, more severe clinical signs than grade 3, with more than 10 cells in the anterior chamber with or without the formation of hypopyon (ie: accumulation of inflammatory cells in the anterior chamber); grade 5, intense inflammatory reaction, fibrin formation in the anterior chamber, and total seclusion of the pupil (ie: pupil that does not react to light due to fibrin clot). Scoring was performed by a masked investigator.

### Cytokine and Chemokine Analysis in Aqueous Humor

Multiplex ELISA assay (Milliplex Map Kit, Millipore, Saint-Quentin en Yvelines, France), was performed on rat aqueous humor at 6 and 24 hours after LPS injection according to the manufacturer’s instructions. The following inflammatory mediators were measured: interleukin-1beta (IL-1β), IL-2, IL-4, IL-5, IL-6, IL-10, IL-13, IL-17, IL-18, tumor necrosis factor-alpha (TNF-α), interferon-gamma (IFN-γ), vascular endothelial growth factor (VEGF); chemokines: monocyte chemotactic protein-1 (MCP-1), macrophage inflammatory protein-1alpha (MIP-1α), regulated upon activation normal T cell expressed and secreted (RANTES), interferon-inducible protein-10 (IP-10), growth related oncogene (GRO/KC). Detection thresholds of the kits were 1 to 10 pg/ml.

### Immunofluorescence

Eyes were snap frozen in Tissue-Tek OCT-compound (Bayer Diagnostics, Puteaux, France). Cryostat sections (10 µm) were incubated with primary antibodies, further incubated with the corresponding secondary antibodies (Invitrogen, Cergy-Pontoise, France) and stained with 4′, 6-Diamidino-2-Phenyl-Indole (DAPI, 1∶3000, Sigma-Aldrich). Control sections were stained without primary antibodies. Images were taken using a fluorescence microscope (Olympus BX51, Rungis, France) equipped with a CCD camera (Olympus DP70). The following primary antibodies were used: mouse monoclonal anti-iNOS (dilution 1∶75, Santa Cruz Biotechnology, CA, USA), rabbit polyclonal anti-ionized calcium binding adaptor molecule-1 (anti-IBA-1; dilution 1∶400, Wako, Richmond, USA) a specific marker for microglia/macrophages [26].

### Quantification of Activated IBA-1 Stained Cells

To quantify ocular cell infiltration/activation and the effect of aldosterone or spironolactone treatment on ocular inflammation, all round activated IBA-1 stained cells were counted on entire ocular cross sections (2 sections per eye) at the optic nerve level, as previously described [25].

### MR and GR Immunohistochemistry

GR and MR were evaluated at 24 hours after LPS injection by immunohistochemistry on eye paraffin sections as previously described [14]. Negative controls were performed without primary antibodies. The following antibodies were used: mouse monoclonal anti-MR 6G1 (1∶100, kindly provided by C. Gomez-Sanchez, Division of Endocrinology, University of Mississippi Medical Center, Jackson, MS), rabbit anti-GR (1∶2000, Santa Cruz, Heidelberg, Germany), biotinylated horse anti-mouse IgG BA2000 (1∶250, Vector) and biotinylated goat anti-rabbit IgG BA1000 (1∶500, Vector).

### Reverse Transcription and Real-time PCR

Total RNA was isolated from iris/ciliary body using RNeasy plus Mini Kit (Qiagen, Courtaboeuf, France) for real-time PCR analysis of MR, GR, 11β-HSD1 and 11β-HSD2 expression as previously described [14]. RNA was also extracted from cells collected in the aqueous humor at 24 hours to quantify iNOS expression in infiltrating cells. The 18S was used as internal control. Table 1 shows sequences of primers.

### Corticosterone ELISA

A corticosterone acetylcholinesterase competitive EIA assay (Cayman, MI, USA) was performed on rat aqueous humor according to the manufacturer’s instructions. Absorbance was measured with a multi-well plate reader (Benchmark Plus Microplate Spectrophotometer System, Bio-Rad Laboratories, California, USA).

### Statistics

Data were expressed as means ± SEM. n is the number of rats. Statistical analysis was made using the Graphpad Prism5 program (Graphpad Software, San Diego, CA, USA). Clinical scoring was analyzed using the non-parametric Kruskal Wallis test followed by Dunn’s multiple comparison test. Other data were analyzed with one-way ANOVA test followed by Bonferroni’s comparison. *P*<0.05 deemed significant.

**Table 1:** Real-time PCR primers.

Gene	SYBR Green Primers (rat)
18S sense	5′-AAG TCC CTG CCG TTT GTA CAC A-3′
18S antisense	5′-GAT CCG AGG GCC TCA CTA AAC-3′
GR sense	5′-AAC ATG TTA GGT GGG CGT CAA-3′
GR antisense	5′-GGT GTA AGT TTC TCA AGC CTA GTA TCG-3′
MR sense	5′-GGC TAC CAC AGT CTC CCT GA-3′
MR antisense	5′-ACG TTG ACA ATC TCC ATG TAG-3′
11β-HSD1 sense	5′-GAA GAA GCA TGG AGG TCA AC-3′
11β-HSD1 antisense	5′-GCA ATC AGA GGT TGG GTC AT-3′
11β-HSD2 sense	5′-TGC TGG CTG GAT CGC GTT GTC-3′
11β-HSD2 antisense	5′-CAC AGT GGC CAG CAC GGT GAA-3′
iNOS sense	5′-CTC GGA GGT CCA CCT CAC TGT-3′
iNOS antisense	5′-GGT TAT TGA TCC AAG TGC TGC-3′

## Results

### Down-regulation of MR and 11β-HSD2 Expression in Iris/ciliary Body During Uveitis

**Figure 1:**
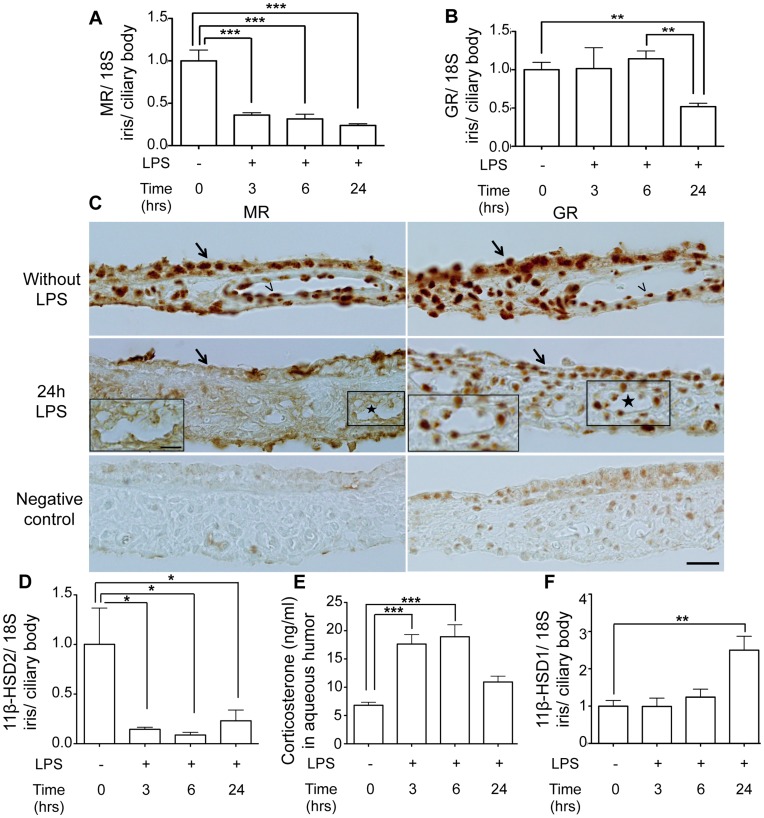
MR, GR, 11β-HSD1, 11β-HSD2 expression in iris/ciliary body and corticosterone concentration in aqueous humor during EIU. A–B: Time-course of MR (A) and GR (B) mRNA expression (real-time PCR). After LPS injection, down-regulation of GR was observed only at 24 hours, whilst MR expression was down-regulated already at 3 hours. The 18S was used as housekeeping gene. Results in EIU are relative to those without LPS injection. Data are means ± SEM; n = 6 rats per conditions; **p<0.01, ***p<0.001. **C:** Immunohistochemistry of MR and GR in iris sections. In normal iris rat, MR and GR are localized in the nuclei of the iris epithelial cells (arrow), endothelial cells (arrowhead) and probably in other resident cells of the iris stroma. Twenty-four hours after LPS injection, MR and GR expression signals were reduced in uveitis eyes (star: vessels). Negative controls are signals yielded in the absence of primary antibody. Scale bar = 40 µm; 20 µm for insert. **D–F:** Time-course of 11β-HSD2 (D), 11β-HSD1 (F) expression (real-time PCR) and corticosterone concentration in aqueous humor (E). LPS injection induced an early (3 hours) down-regulation of 11β-HSD2 transcripts and a late up-regulation of 11β-HSD1 transcripts at 24 hours compared to rats without LPS injection. The 18S was used as housekeeping gene. Results in EIU are relative to those without LPS injection. Corticosterone concentration in aqueous humor increases at 3 and 6 hours after LPS injection. Data are means ± SEM; n = 6 rats per conditions; *p<0.05, **p<0.01, ***p<0.001.

Real-time PCR analyses show that MR mRNA is expressed in rat iris/ciliary body (Fig. 1A, without LPS). As early as 3 hours after LPS injection, a major down-regulation of MR expression was observed, that was sustained at 6 and 24 hours (Fig. 1A). Reduction in MR levels was specific for this receptor, as the closely related glucocorticoid receptor (GR) transcripts levels were not altered in the early phase of EIU (3 and 6 hours); down-regulation of GR mRNA was observed only 24 hours after onset of EIU (Fig. 1B). Immunolocalization experiments extended these observations at the protein level (Fig. 1C). The MR and the GR are expressed in the iris epithelial and endothelial cell nuclei and probably in other resident cells of the iris stroma (Fig. 1C, without LPS). Twenty-four hours after LPS injection, MR and GR were down-regulated (Fig. 1C, 24 h LPS). The lower GR staining may be due to swollen tissues, in addition to the lower expression of GR inferred from the low GR transcript levels at 24 hours.

Down-regulation of transcripts encoding for the glucocorticoid-inactivating enzyme 11β-HSD2 (corticosterone to 11dehydrocorticosterone in rats) was parallel to the down-regulation of the MR (Fig. 1D); corticosterone concentration in aqueous humor was enhanced 3 and 6 hours after LPS injection (Fig. 1E). In contrast, the mRNA levels of enzyme 11β-HSD1 driving the opposite reaction (11dehydrocorticosterone to corticosterone) were higher 24 hours after LPS challenge (Fig. 1F).

These results led us to question the role of the MR in uveitis.

### MR Activation Limits the Severity of Uveitis

**Figure 2:**
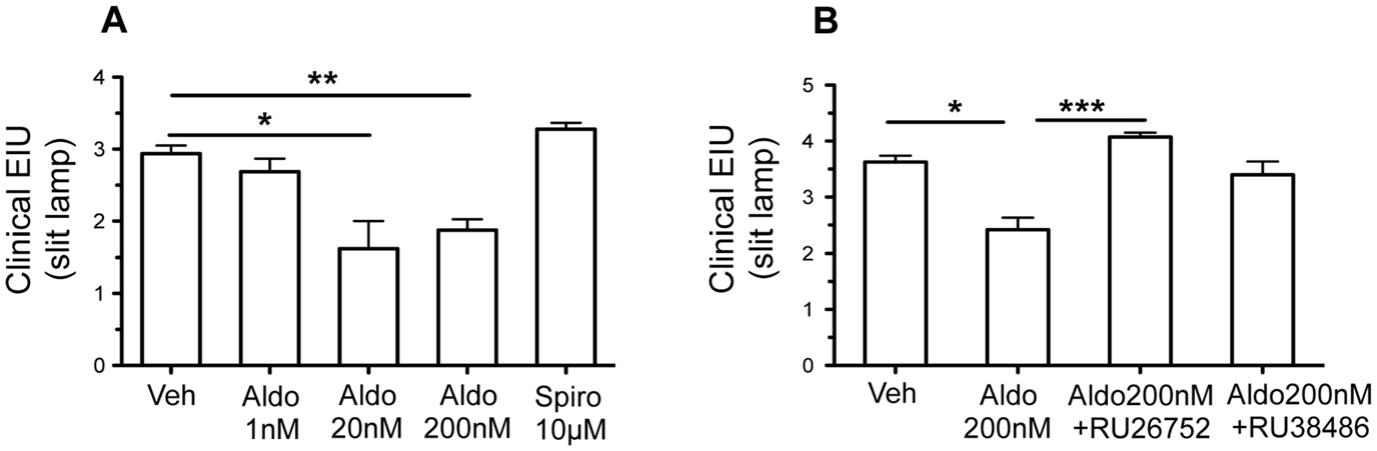
Effects of intravitreal injection of aldosterone or spironolactone on clinical EIU. Clinical scores of the severity of EIU were determined 24 hours after induction of EIU. Steroids were injected IVT in each eye, simultaneously to footpad LPS injection; 24 hours later, both eyes were evaluated, and scores of each eye were gathered as the clinical score of the animal. **A:** Intravitreal injection (IVT) of aldosterone (aldo; 1, 20 or 200 nM) or spironolactone (spiro; 10 µm): aldosterone (20–200 nM) induced a significant reduction of the clinical severity of EIU, compared to vehicle (Veh) IVT. Spironolactone did not modify the severity of EIU. Data are mean ± SEM; n = 8 rats per condition. **B:** Intravitreal injection of aldosterone (200 nM) alone or associated with the MR antagonist RU26752 (20 µM) or with the GR antagonist RU38486 (20 µM): benefit of aldosterone injection was confirmed, which was blunted in the presence of RU26752. There was no statistical difference between aldosterone+RU38486 IVT and vehicle injection or aldosterone IVT. Data are mean ± SEM; n = 10 rats per condition; *p<0.05, **p<0.01, ***p<0.001.

Clinically, uveitis is characterized by vasodilation of the iris vessels, infiltration of inflammatory cells in the eye and proteins in aqueous humor that witness rupture of ocular barriers. Clinical scoring was performed at 24 hours, the peak of inflammation in our model [25]. Aldosterone, injected in the eyes concomitant to LPS challenge, induced a significant and dose-dependent reduction of the clinical intensity of EIU as compared to vehicle-injected rats (Fig. 2A); maximal protective effect of aldosterone was observed at 20–200 nM. Aldosterone improvement of ocular inflammation was suppressed when the hormone was co-injected with the MR antagonist RU26752, indicating that the anti-inflammatory protecting effect of aldosterone is MR-mediated (Fig. 2B). At this concentration, aldosterone may also activate the GR as inferred from the intermediate clinical score obtained by aldosterone associated with the GR antagonist RU38486 (Fig. 2B). None of these antagonists alone significantly modified the clinical score of uveitis (mean ± SEM: Vehicle: 3.6±0.2; RU26752∶4.0±0.1; RU38486∶3.7±0.3; n = 5 rats per conditions).

In this pathological context, MR antagonism might be deleterious. To test this hypothesis, we injected spironolactone (10 µM) intravitreously, concomitantly with LPS injection in footpad, to block (or limit) MR activation by endogenous ligands (aldosterone or glucocorticoids) (Fig. 2A). However the MR antagonist did not significantly increase the clinical severity of the disease.

All further analyses were performed with aldosterone 200 nM and spironolactone 10 µM.

### Aldosterone-MR Activation Reduces Pro-inflammatory Mediators

**Figure 3:**
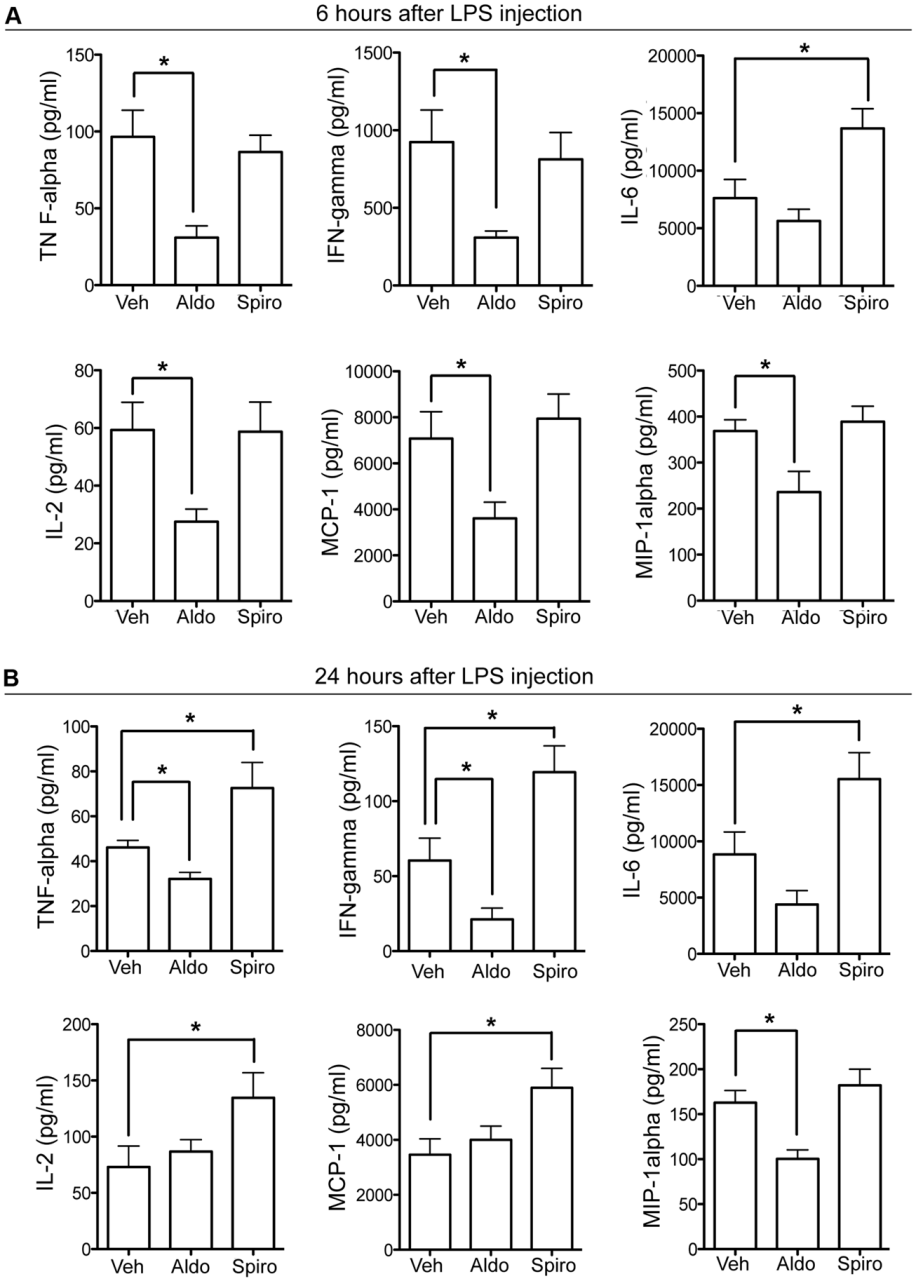
Cytokine/chemokine concentrations in aqueous humor. Cytokine/chemokine concentrations in aqueous humor were measured by multiplex ELISA 6 or 24 hours after LPS injection associated to vehicle (Veh) or aldosterone (Aldo) or spironolactone (Spiro) intravitreal injection. **A:** At 6 hours, TNF-α, IFN-γ, IL-2, MCP-1, and MIP-1α concentrations were significantly reduced in inflamed eyes with injection of aldosterone, compared to vehicle-injected inflamed eyes; spironolactone enhanced IL-6 concentrations in rats with EIU. Data are mean ± SEM; n = 5 rats per condition. **B:** At 24 hours, Aldosterone significantly reduced TNF-α, IFN-γ and MIP-1α concentrations; spironolactone increased TNF-α, IFN-γ, IL-6, IL-2 and MCP-1 concentrations. Data are mean ± SEM; n = 8 rats per condition. *p<0.05.

To assess the anti-inflammatory role of aldosterone in EIU, we determined the effect of aldosterone injection on chemokines and cytokines production in aqueous humor. In the early phase of EIU (i.e. 6 hours), aldosterone significantly decreased several pro-inflammatory mediators: TNF-α, IFN-γ, IL-2, MCP-1, MIP-1α as compared to vehicle injection while IL-6 levels were not modified (Fig. 3A). At 24 hours the aldosterone-induced reduction of TNF-α, IFN-γ and MIP-1α was sustained (Fig. 3B). There was no significant effect on other tested cytokines (IL-1β, IL-18, IL-4, IL-5, IL-10, IL-13, IL-17) or chemokines (RANTES, IP-10, GRO-KC) (data not shown). In contrast, intravitreal injection of spironolactone led to a significant increase in IL-6 at 6 hours (Fig. 3A) and a significant increase in TNF-α, IFN-γ, IL-6, IL-2 and MCP-1 at 24 hours (Fig. 3B).

It can be noticed that cytokines/chemokines concentrations were higher at 6 hours than at 24 hours, consistent with previous studies showing a peak of pro-inflammatory cytokines about 3–6 hours after LPS injection [27], [28].

These results show that MR activation by aldosterone exerts anti-inflammatory effects in EIU through early reduction of pro-inflammatory cytokines in ocular fluids. Of interest, these variations occurred at a time when circulating inflammatory cells have not yet penetrated into the eye and thus could correspond to the secretion of cytokines by ocular activated resident cells.

### Aldosterone/MR Influences Microglia Activation

**Figure 4:**
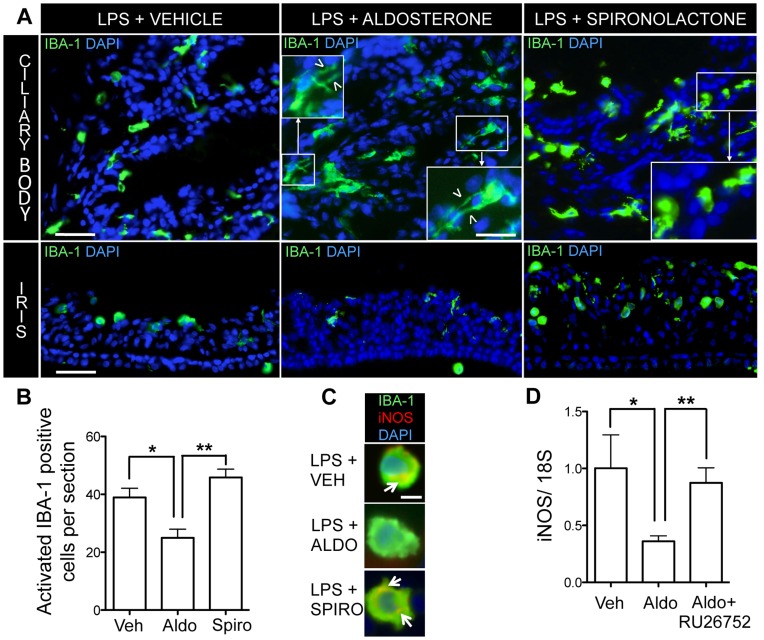
Aldosterone reduces activation of microglia/macrophages in EIU. A: Immunofluorescence detection of IBA-1 microglia/macrophages on sections of inflamed eyes injected with vehicle, aldosterone or spironolactone. Twenty-four hours after LPS injection, in vehicle and spironolactone-injected eyes, many microglial cells had a round amoeboid shape attesting for their activation in the iris/ciliary body. By contrast, in aldosterone-injected eyes, more microglial cells had a resting dendritic shape with long branchings (white arrowheads). Merge images of IBA-1 (green) and DAPI (blue); scale bar: ciliary body: 40 µm; 25 µm for insert; iris: 60 µm. **B:** Quantification of activated IBA-1 microglia/macrophages on sections of inflamed eyes that were injected with vehicle, aldosterone or spironolactone. Twenty-four hours after LPS injection, less number of round activated IBA-1 positive cells was found in eyes sections with injection of aldosterone (Aldo) vs vehicle (Veh) or spironolactone (Spiro). Data are expressed as means ± SEM of 2 sections/rat; n = 4 rats per condition; *p<0.05, **p<0.01. **C:** Immunofluorescence of iNOS on sections of inflamed eyes injected with vehicle, aldosterone or spironolactone. Twenty-four hours after LPS injection, few round IBA-1 positive macrophages/microglia expressed iNOS in vehicle and spironolactone-injected eyes (colocalization: yellow, arrow). By contrast, iNOS signal was reduced in IBA-1 positive macrophages/microglia in aldosterone-treated eyes. Merge image of IBA-1 (green), iNOS (red) and DAPI (blue); scale bar: 10 µm; n = 4 rats per condition. **D:** iNOS mRNA expression (real-time PCR) in cells infiltrating the aqueous humor in EIU. In EIU, inflammatory cells (neutrophils and macrophages) accumulate in aqueous humor. In cells collected in aqueous humor from EIU eyes, iNOS mRNA expression was significantly reduced by aldosterone (Aldo) IVT compared to vehicle (Veh) IVT. This effect was prevented by the co-administration of aldosterone with the MR antagonist RU26752. The 18S was used as housekeeping gene. Results are relative changes compared to vehicle-injected eyes. Data are means ± SEM; n = 4–5 rats per condition; *p<0.05.

To evaluate the effect of aldosterone on microglia activation and macrophages recruitment, we used the anti-ionized calcium binding adaptor molecule 1 (IBA-1) on cryostat sections. Round-shaped IBA-1 positive cells represent activated cells while ramified ones are resting cells. In aldosterone-injected eyes, most microglial cells in iris/ciliary body showed a resting ramified shape with ramifications as compared to round-shaped IBA-1 positive cells in vehicle or spironolactone-injected eyes (Fig. 4A). The number of round activated IBA-1 positive cells was significantly decreased in aldosterone-injected eyes as compared to vehicle or spironolactone-injected ones (Fig. 4B). Co-labeling of IBA-1-positive cells with iNOS showed that iNOS was not expressed by microglia in aldosterone-treated eyes, whilst few round microglia expressed iNOS in spironolactone or vehicle-treated eyes (Fig. 4C). Further, in infiltrating cells collected from aqueous humor of eyes with uveitis, iNOS mRNA expression was significantly reduced by aldosterone, compared to vehicle (Fig. 4D). This effect was prevented by the co-administration of aldosterone plus RU26752 (MR antagonist) (Fig. 4D), demonstrating the anti-inflammatory role of MR pathway.

### In EIU, Aldosterone Restores MR Expression in Iris/ciliary Body

**Figure 5:**
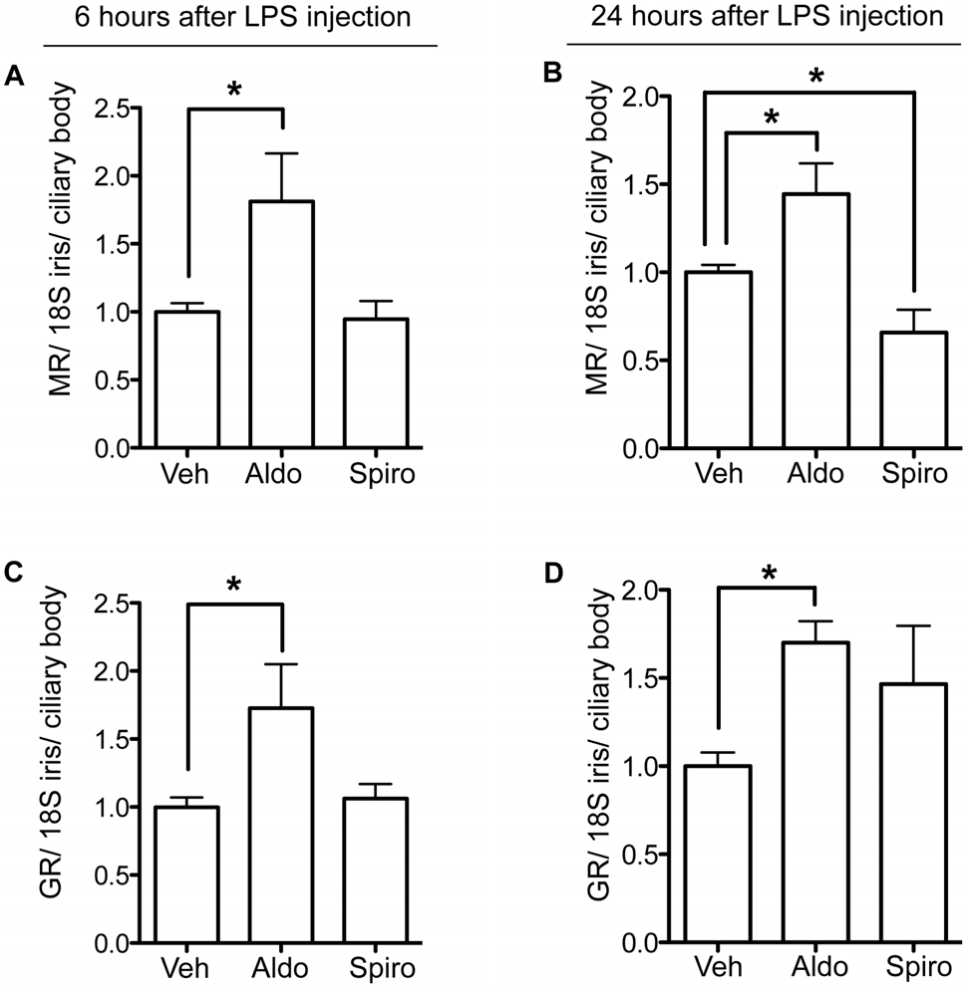
MR and GR mRNA expression after intravitreal injection of aldosterone or spironolactone in the eye of LPS-injected rats. EIU was associated to down-regulation of MR and GR (Fig. 1). **A–B:** MR expression in iris/ciliary body (real-time PCR): In the inflamed eye, intravitreal injection of aldosterone (Aldo) increased MR transcripts at 6 hours (A) and 24 hours (B) in EIU vs vehicle-injected eyes (Veh). Intravitreal injection of spironolactone (Spiro) decreased MR transcripts compared to vehicle-injected rats at 24 hours (B) and had no effect at 6 hours (A). **C–D:** GR expression in iris/ciliary body (real-time PCR): In the inflamed eye, intravitreal injection of aldosterone increased GR transcripts at 6 hours (C) and 24 hours vs vehicle-injected eyes (D). GR expression was not statistically modified by spironolactone. The 18S was used as housekeeping gene. Results are relative values of aldo/spiro relative to vehicle injection. Data are means ± SEM; n = 5–7 rats per condition; *p<0.05.

Aldosterone IVT restored (at least partially) the MR expression that had been reduced by LPS during EIU (Fig. 1): improvement was evidenced 6 hours after onset of EIU (an increment that was sustained at 24 hours) (Fig. 5A, B). GR expression was also increased after aldosterone IVT injection at 6 hours and 24 hours (Fig. 5C, D). On the opposite, intravitreal injection of spironolactone decreased MR transcripts compared to vehicle-injected rats at 24 hours (Fig. 5B) and had no effect at 6 hours (Fig. 5A).

## Discussion

In humans as well as in animal models, long-term inappropriate or excessive MR activation, due to chronic ligand increase (aldosterone or glucocorticoids) or receptor overexpression and/or activity, was found to promote inflammation and oxidative stress markers in kidney and cardiovascular pathologies [29]. Of note, deleterious effects of aldosterone require association of MR activation to a pathological context or to additional stress, such as high sodium diet and uninephrectomy. Of major clinical relevance, it was demonstrated that MR antagonism limits the progression of cardiovascular end-organ damage [4], [5].

The retina and the choroid are now emerging as novel mineralocorticoid-sensitive tissues, as the MR pathway has recently been evidenced in normal and diseased eye [14], [15], [30]. We previously showed that the retina is a mineralocorticoid target and that the MR pathway regulates ion and water channels expression in Müller glial cells, contributing to the fluid homeostasis of the retina [14]. We also demonstrated that MR pathway controls choroidal vascular bed relaxation and is involved in the pathogenesis of central serous chorioretinopathy, a human ocular disease [15]. The group of Wilkinson-Berka identified MR involvement in retinal vascular pathology in a rat model of oxygen-induced retinopathy [30], as aldosterone favored pathological angiogenesis of retinal vessels and participated to retinal inflammation in this model.

The MR may also be active in the anterior segment of the eye, in particular in the iris and ciliary body. Its expression has been documented in several species: GR, MR and 11β-HSD1 and 2 have been found in the ciliary body epithelium of the rabbit eye [17]; MR and 11β-HSD2 are highly expressed in the ciliary body epithelium of the rat and human eye [16]. Here we document MR expression in the iris of rat eye. MR is therefore found in all cells that constitute the blood-aqueous barrier, raising the question of its role in an acute inflammatory state, such as uveitis.

In EIU, a rat model of uveitis, LPS activates macrophage/microglia [23], [31]. Both infiltrating and resident ocular cells produce cytokines and iNOS. While iNOS mRNA was not detected in the control eye (without LPS injection), it increased from 2 to 24 hours after LPS injection [32], [33]. The local cytokines may originate from resident activated cells or from systemic production reaching the damaged eye [34]. TNF-α is the most important cytokine in the pathogenesis of EIU. It has been shown that the mRNA levels of TNF-α are enhanced in the eye, as early as 3 hours after LPS injection [22]. Most cytokines involved in EIU achieved peak mRNA level in iris/ciliary body about 3–6 hours after LPS injection [28]. Our findings are consistent with these kinetics. The lower values of IFN-γ found in our study at 24 h may depend on the half-life of the cytokine in aqueous humor or on individual variations, as reported by De Vos et al [34].

In sharp contrast with the bulk of literature reports on chronic deleterious effects of aldosterone, we found that relatively short-term aldosterone administration is anti-inflammatory in the inflamed eye, in the context of EIU, an acute ocular inflammation model. Although clinical scoring is subjective, its improvement by aldosterone is strengthened by convergent data including cytokine profiles in ocular media, cells counts in ocular tissues, microglia activation in the iris/ciliary body and iNOS expression in inflammatory cells accumulating in aqueous humor.

Recent studies questioned the role of the MR in an infectious context. In dog sepsis, specific MR agonists increased survival when given preventively [35]. It was also observed that mortality was enhanced by spironolactone during infectious colitis in two rodents model and in humans [36]. In neutrophils, functional MR was reported to mediate anti-inflammatory effects via inactivation of nuclear factor-kappa B (NF-κB); indeed aldosterone strongly diminished NF-κB dependant TNF-α production in IL-8 stimulated neutrophils, resulting in reduced neutrophil adhesion to endothelial cells [37].

MR activation may also be beneficial in non-infectious situations. Cardiac aldosterone production was shown to prevent the reduction in capillary density in diabetic mice [38]. Pro-angiogenic effects of aldosterone were also inferred from improved neovascularization following limb ischemia in mice [39]. Thus, depending on the cellular and pathological context, aldosterone/MR signaling may exert opposite effects, either deleterious or beneficial.

While anti-inflammatory effects of glucocorticoids and GR pathway have been largely documented, this study brings new data demonstrating that the aldosterone/MR pathway exerts also anti-inflammatory effects. The beneficial effect of aldosterone in uveitis was observed at relatively high dose (200 nM) that may activate both MR and GR. However, *in vivo* the exact dose that efficiently reaches the iris/ciliary body after intravitreal injection cannot be ensured. The fact that the co-injection of aldosterone with the MR antagonist clearly prevents the anti-inflammatory effect of aldosterone/MR pathway reinforces the idea that the MR pathway is involved in the observed effect. Aldosterone may also activate the GR as inferred from the intermediate clinical score obtained by aldosterone plus RU38486 treatment. Indeed, the GR also has comparable (but low) affinity for binding aldosterone and glucocorticoids (12 nM); However the ED_50_ (Effective Dose 50) for transactivation activity of the GR occupied by dexamethasone is about 15 nM and 120 nM with aldosterone [40]. Moreover, MR can form homodimers or heterodimers with the GR with distinct transcriptional activity [41] and a series of downstream events also influence mineralocorticoid selectivity (co-activators and tissue-specific transcription factors, post-translational processing of the MR [2], [42]…) Any of these elements may be altered in pathology. Very limited information is available yet on these pathophysiological aspects.

The mechanism at the origin of the beneficial effect of aldosterone in uveitis is currently unknown; we propose that it may be related to its ability to prevent the uveitis-induced MR down-regulation, at variance with situations where the MR is inappropriately activated. In the early phase of uveitis (3 hours), there is a series of changes that lead to less activation of MR and of the opposite over-activation of the GR pathway; although GR mRNA levels are unchanged, 11β-HSD2 mRNA is diminished, a finding coherent with the rise in corticosterone in aqueous humor. This result is consistent with the high cortisol levels measured in aqueous humor of patients with uveitis [43]. Corticosterone in aqueous humor may originate from adrenal production as the consequence of the stress induced by uveitis. It may also be the result of the reduced local 11β-HSD2 activity.

Thus, at the early phase of uveitis, the MR/GR balance is shifted towards GR activity. Aldosterone injection restores (at least partially) MR signaling and prevents the MR/GR imbalance.

MR down-regulation was evidenced in vivo as early as 3 hours after LPS injection, i.e. at a time when no cells have infiltrated the eye tissues [22], showing that MR down-regulation is associated to the onset of ocular inflammation. Interestingly, in human retinal and choroidal endothelial cells stimulated by LPS, a transcriptomic analysis had reported reduced MR expression [44]. Moreover, in brain microglial cells, GR and MR are also down-regulated by LPS in a time-dependent manner, with an earlier decrease in MR than in GR, suggesting that MR/GR pathways are involved in the immunosuppressive role of endogenous steroids in these cells [45].

We have no straightforward explanation for the aldosterone-dependent down-regulation of MR and GR in EIU, and limited literature data document such ligand- dependent regulation. One can speculate that GR and MR expression may depend on pathways that are activated during inflammation such as NFκB.

In conclusion, our results show that MR intervenes in the very early events of EIU and that aldosterone is anti-inflammatory in this pathological context. Different glucocorticoids with different MR affinity may therefore exert differential effects in uveitis. Future clinical studies should evaluate this possibility in humans.

## Supporting Information

Figure S1 **Eye drawing shows the uveal tract (red), the middle layer of the eye, formed by the choroid, iris and ciliary body.** These tissues are targets of ocular inflammation called uveitis.
(TIF)
