# Supplementary figures and images for: The Aldosterone-Mineralocorticoid Receptor Pathway Exerts Anti-Inflammatory Effects in Endotoxin-Induced Uveitis
Source: PLoS One. 2012 Nov 9;7(11):e49036. doi: 10.1371/journal.pone.0049036 (PMC3494666; doi:10.1371/journal.pone.0049036)

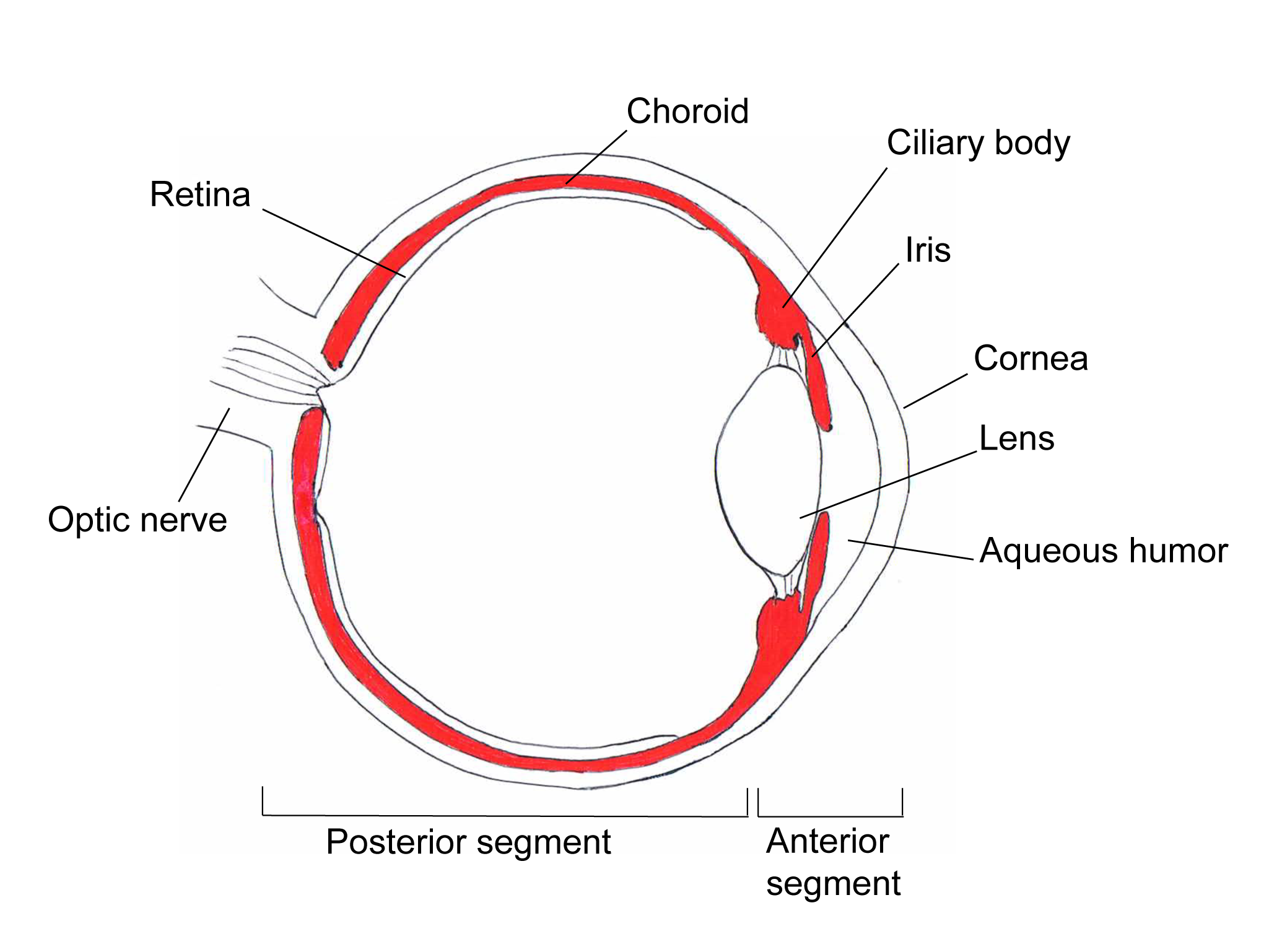

Supplement: Figure S1 — Eye drawing shows the uveal tract (red), the middle layer of the eye, formed by the choroid, iris and ciliary body. These tissues are targets of ocular inflammation called uveitis. (TIF) [file pone.0049036.s001.tif]
